# Challenges in the clinical management of rare diseases and center-based multidisciplinary approach to creating solutions

**DOI:** 10.1007/s00431-025-06101-z

**Published:** 2025-04-05

**Authors:** D. Gunes, M. Karaca, A. Durmus, B. Ak, N. Aktay Ayaz, Z. U. Altınel, A. D. Aslanger, F. Atalar, M. C. Balci, L. Bilgin, F. Darendeliler, D. Demirkol, O. Durmaz, A. Gedikbasi, E. Inan Balci, E. Z. Ince, S. G. Karadag, G. Keskindemirci, K. Nisli, M. Ozcetin, A. Somer, A. Unuvar, M. Uysalol, E. Yildiz, Z. N. Yuruk Yildirim, M. Demirkol, G. F. Gokcay

**Affiliations:** 1 Department of Rare Diseases, Institute of Child Health, Istanbul University, Istanbul, Turkey; 2 Division of Nutrition and Metabolism, Department of Pediatrics, Istanbul Faculty of Medicine, Istanbul University, Istanbul, Turkey; 3 Division of Pediatric Rheumatology, Department of Pediatrics, Istanbul Faculty of Medicine, Istanbul University, Istanbul, Turkey; 4 Division of Pediatric Allergy and Immunology, Department of Pediatrics, Istanbul Faculty of Medicine, Istanbul University, Istanbul, Turkey; 5 Department of Medical Genetics, Istanbul Faculty of Medicine, Istanbul University, Istanbul, Turkey; 6 Division of Neonatology, Department of Pediatrics, Istanbul Faculty of Medicine, Istanbul University, Istanbul, Turkey; 7 Division of Pediatric Endocrinology, Department of Pediatrics, Istanbul Faculty of Medicine, Istanbul University, Istanbul, Turkey; 8 Division of Pediatric Intensive Care Unit, Department of Pediatrics, Istanbul Faculty of Medicine, Istanbul University, Istanbul, Turkey; 9 Division of Pediatric Gastroenterology and Hepatology, Department of Pediatrics, Istanbul Faculty of Medicine, Istanbul University, Istanbul, Turkey; 10 Department of Pediatric Basic Sciences, Institute of Child Health, Istanbul University, Istanbul, Turkey; 11 Division of Social Pediatrics, Department of Pediatrics, Istanbul Faculty of Medicine, Istanbul University, Istanbul, Turkey; 12 Division of Pediatric Cardiology, Department of Pediatrics, Istanbul Faculty of Medicine, Istanbul University, Istanbul, Turkey; 13 Department of Pediatrics, Istanbul Faculty of Medicine, Istanbul University, Istanbul, Turkey; 14 Division of Pediatric Infectious Diseases, Department of Pediatrics, Istanbul Faculty of Medicine, Istanbul University, Istanbul, Turkey; 15 Division of Pediatric Hematology and Oncology, Department of Pediatrics, Istanbul Faculty of Medicine, Istanbul University, Istanbul, Türkiye; 16 Division of Pediatric Emergency, Department of Pediatrics, Istanbul Faculty of Medicine, Istanbul University, Istanbul, Turkey; 17 Division of Pediatric Neurology, Department of Pediatrics, Istanbul Faculty of Medicine, Istanbul University, Istanbul, Turkey; 18 Division of Pediatric Nephrology, Department of Pediatrics, Istanbul Faculty of Medicine, Istanbul University, Istanbul, Turkey

**Keywords:** Multidisciplinary approach, Rare diseases, Specialist education, Solution proposals, Türkiye

## Abstract

The diagnosis and treatment of rare diseases present significant global challenges. This study aimed to identify the difficulties faced by specialists in the diagnosis and management of rare diseases, as well as to gather their recommendations for potential solutions. An expert committee specializing in inborn metabolic disease and genetics developed a comprehensive survey, which was then distributed online to professionals working with rare diseases. A total of 21 specialists actively engaged in the management of rare diseases participated in the survey. All participants acknowledged the substanstial significant diagnostic challenges associated with rare diseases, with 86% indicating that these diagnostic challenges negatively affect their clinical practice. The primary obstacles encountered in the diagnosis and follow-up of rare diseases were low awareness, a lack of a multidisciplinary approach, insufficient numbers of specialists and inadequate infrastructure, limited newborn screening programs, challenges in accessing treatment, and insufficient psychosocial support. All participants emphasized the need for a multidisciplinary approach in the management of rare diseases. Proposed solutions included enhanced training for healthcare professionals, the establishment of multidisciplinary teams and diagnostic algorithms, the regular convening of councils and meetings, and the establishment of robust registries. While all participants rated their own clinical experience as proficient in diagnosing and treating rare diseases, the establishment of multidisciplinary teams was the most frequently suggested area for improvement.

*Conclusion*: Addressing the challenges in the diagnosis, treatment, and monitoring of rare diseases requires a multifaceted approach, including raising awareness, enhancing patient services, developing robust research and improving the infrastructure, establishing multidisciplinary care frameworks, and implementing preventive medicine and social policies.

**Table:** 

**What is Known:** •* It is estimated that over 300 million people globally are living with one or more rare diseases. The process of diagnosis, treatment, and follow-up of rare diseases involves significant global challenges.*
**What is New:** • *In our study, the difficulties encountered by specialists in the diagnosis and treatment of rare diseases in Türkiye and solution suggestions are presented. This is the first study on this subject in Türkiye.*

**What is Known:**

•* It is estimated that over 300 million people globally are living with one or more rare diseases. The process of diagnosis, treatment, and follow-up of rare diseases involves significant global challenges.*

**What is New:**

• *In our study, the difficulties encountered by specialists in the diagnosis and treatment of rare diseases in Türkiye and solution suggestions are presented. This is the first study on this subject in Türkiye.*

## Introduction

Rare diseases, encompassing over 7000 identified conditions to date, are defined as disorders with a prevalence of less than 1 in 2000 individuals within a World Health Organization (WHO)–defined region of the world [1, 2]. Although rare individually, rare diseases affect a population size equivalent to the third largest country in the world collectively [3, 4]. Currently, it is estimated that over 300 million people globally are living with one or more rare diseases. Seventy percent of these diseases manifest in childhood. Approximately 72% of all rare diseases are attributed to genetic causes [5]. Non-genetic rare diseases include cancers, autoimmune diseases, congenital malformations, and toxic and infectious diseases. The high frequency of consanguineous marriages in our country increases the risk of rare diseases, resulting in an estimated 5 to 6.4 million individuals in Türkiye being affected by these conditions [6].

Inadequate coverage of rare diseases in medical education limited awareness among physicians, a scarcity of specialists dedicated to these conditions, the absence of specific health policies, challenges in accessing laboratory analyses on time, and a lack of multidisciplinary evaluation can collectively contribute to significant delays in the diagnosis of rare diseases. Such diagnostic and treatment delays may result in increased mortality and morbidity. Under the leadership of the Autism, Mental Special Needs, and Rare Diseases Department within the Turkish Ministry of Health and with contributions of all invited stakeholders, the “2023–2027 Rare Diseases Health Strategy Document and Action Plan” has been published [6].

In our country, various training programs on rare diseases are available for healthcare professionals providing valuable opportunities to assess clinicians’ experiences and needs across different disciplines [7]. Istanbul as a major metropolitan city receives immigrants from diverse regions of Turkey and serves as a healthcare hub for patients from across the country. The Istanbul Faculty of Medicine is one of the Turkiye’s leading centers for rare diseases, offering a multidisciplinary approach by bringing together specialists from all relevant fields. For the past 6 years, Istanbul University Istanbul Faculty of Medicine has organized educational meetings on Rare Disease Day to enhance awareness, address diagnostic and treatment challenges, and explore potential solutions. These meetings provide a platform where clinicians from various specialities discuss current developments, existing challenges, and strategies for managing rare diseases within their respective fields. These meetings are planned to improve the visibility of rare diseases by sharing and exchanging knowledge between clinicians, Turkish Ministry of Health officials, patients, and their families, as well as representatives from non-governmental organizations. Prior to the Rare Diseases training meeting in 2023, an online survey was conducted to identify and discuss the challenges pediatric clinicians face in diagnosing and treating rare diseases and to explore the potential solutions. The survey results, evaluated in this study, are expected to highlight the difficulties encountered in the diagnosis and treatment of rare diseases in our country and propose viable solutions. This study represents the first of its kind conducted in Türkiye on this subject.

## Materials and methods

The study included 21 participants from 16 different subspecialties of pediatrics who attended the Rare Diseases Day meeting organized by Istanbul University Istanbul Faculty of Medicine in 2023. Participants were asked to provide and record their age, gender, and specialty. A Turkish-language online survey was administered to gather data on rare diseases, the challenges faced by patients with rare conditions, and potential solutions. Designed by a committee of inborn error of metabolism (IEM) experts and geneticists based on literature data and field observations, this survey consisted of both open-ended and multiple-choice questions to obtain detailed responses to specific challenges encountered in the diagnosis and treatment of rare diseases and to provide suggestions for solving these problems. In addition to multiple-choice questions, a Likert-type scale was also used for some questions (Table 1). This study was approved by the Ethics Committee of Istanbul University Istanbul Faculty of Medicine (Ethics Committee number: 2024/1662).

**Table 1:** Survey questions

Number	Question	Question type	Response options	Participants (*n*)
1	Based on the information on rare diseases with a frequency of less than 1 in 2000 in the population, what percentage of patients in your clinical practice have been diagnosed with these conditions?	Multiple-choice	a) 0–20% b) 21–40% c) 41–60% d) 61–80% e) 81–100%	21
2	What are the three rare diseases you encounter most frequently in your clinical practice?	Open-ended	-	21
3	In your clinical practice, do you agree that there are diagnostic difficulties associated with rare diseases?	Likert scale	a) Strongly agree b) Agree c) Undecided d) Disagree e) Strongly disagree	21
4	Does the difficulty of diagnosing rare diseases affect your work in your clinical practice?	Likert scale	a) Strongly agree b) Agree c) Undecided d) Disagree e) Strongly disagree	21
5	Do you agree that rare diseases should have a separate registration system?	Likert scale	a) Strongly agree b) Agree c) Undecided d) Disagree e) Strongly disagree	21
6	Do you agree that a multidisciplinary approach is essential for the management of rare diseases?	Likert scale	a) Strongly agree b) Agree c) Undecided d) Disagree e) Strongly disagree	21
7	Do you believe there are challenges in implementing a multidisciplinary approach to the management of rare diseases in your clinical practice?	Likert scale	a) Strongly agree b) Agree c) Undecided d) Disagree e) Strongly disagree	21
8	Could you provide three suggestions for improving the multidisciplinary approach to rare diseases in your clinical practice?	Open-ended	-	21
9	List three major problems in the diagnosis of rare diseases in order of importance	Open-ended	-	21
10	List three major problems in the treatment of rare diseases in order of importance	Open-ended	-	21
11	What are the strengths or advantages of your clinic in diagnosing and treating rare diseases?	Open-ended	-	21
12	What are the limitations or challenges faced by your clinic in diagnosing and treating rare diseases?	Open-ended	-	21
13	What strategies can be implemented to enhance the diagnosis and treatment of rare diseases?	Open-ended	-	21
14	What are your expectations from other teams in the clinic regarding the diagnosis and treatment of rare diseases?	Open-ended	-	21
15	How would you assess the proficiency of our institution in accurately diagnosing rare diseases?	Likert scale	a) Very good b) Good c) Neither good nor bad d) Bad e) Very bad	21
16	How would you evaluate your clinic’s capability in administering treatments for rare diseases?	Likert scale	a) Very good b) Good c) Neither good nor bad d) Bad e) Very bad	21
17	What recommendations do you have for advancing the diagnosis and treatment of rare diseases in your clinic?	Open-ended	-	21

## Results

Among the 21 participants, 81% were female, with ages ranging from 36 to 58 years. The participants represented a variety of specialist departments, including pediatric metabolic diseases (*n* = 4), genetics (*n* = 2), pediatric rheumatology (*n* = 2), pediatric gastroenterohepatology (*n* = 1), pediatric endocrinology (*n* = 1), pediatric hematology (*n* = 1), pediatric emergency medicine (*n* = 1), pediatric nephrology (*n* = 1), pediatric allergy and immunology (*n* = 1), pediatric infectious diseases (*n* = 1), pediatric cardiology (*n* = 1), pediatric neurology (*n* = 1), pediatric intensive care (*n* = 1), newborn intensive care (*n* = 1), general pediatrics (*n* = 1), and social pediatrics (*n* = 1). The average professional experience of the participants was 22.4 ± 7.2 years (range: 13–33 years).

**Fig. 1:**
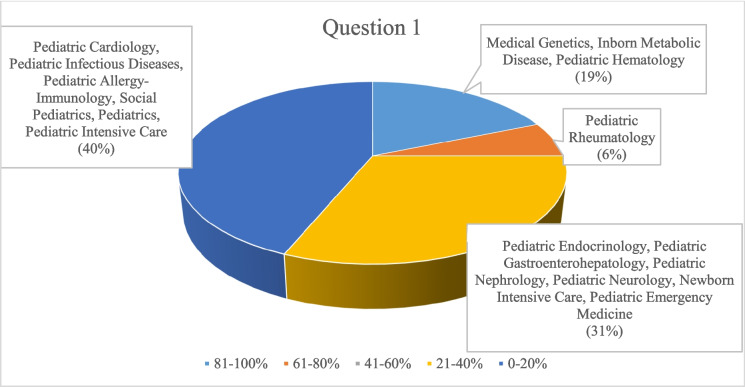
Prevalence rates of rare diseases encountered by participants in their clinical practice

The prevalence rates of rare diseases encountered by participants in their clinical practice are illustrated in Fig. 1.

The responses from participants concerning the rare diseases they most frequently encounter in their clinical practice are detailed in Table 2.

**Table 2:** Rare diseases most frequently encountered by various pediatric subspecialties in their clinical practice

Inborn metabolic disease Medical genetics Pediatric hematology Pediatric rheumatology Pediatric endocrinology Pediatric gastroenterohepatology Pediatric nephrology Pediatric neurology Pediatric emergency Pediatric cardiology Pediatric infectious diseases Pediatric allergy-immunology Social pediatrics Pediatrics Pediatric intensive care Newborn intensive care	Phenylketonuria, biotinidase deficiency, lysosomal storage diseases Neurometabolic diseases, Duchenne muscular dystrophy, Turner syndrome Hemophilia, other clotting factor deficiencies, leukemia Autoinflammatory diseases, vasculitis, chronic arthritis Achondroplasia, congenital adrenal hyperplasia, Noonan syndrome Wilson’s disease, progressive familial intrahepatic cholestasis, biliary atresia Congenital kidney and urinary system anomalies, glomerular diseases, ciliopathies Spinal muscular atrophy, Duchenne muscular dystrophy, congenital myasthenia gravis Bartter syndrome, Dravet syndrome, congenital adrenal hyperplasia Mucopolysaccharidoses, muscle diseases, tuberous sclerosis Acquired immune deficiency syndrome Cystic fibrosis, primary ciliary dyskinesia, primary immunodeficiencies Down syndrome, familial Mediterranean fever, congenital hypothyroidism Henoch Schonlein vasculitis, cerebral palsy, tuberculosis Inborn metabolic diseases, cardiomyopathies, renal genetic diseases Inborn metabolic diseases, multiple congenital anomalies, spinal muscular atrophy

All participants concurred that there are significant diagnostic challenges associated with rare diseases, with 33% strongly agreeing and 67% agreeing. Moreover, 86% of the participants indicated that the diagnostic difficulties adversely impact their clinical work, with 38% strongly agreeing and 48% agreeing. Notably, the pediatric gastroenterology and pediatric emergency departments disagreed with this statement.

Regarding the registration of rare diseases, 86% of the participants advocated for a distinct registration system, while 14% of the participants comprising representatives from pediatric gastroenterohepatology, social pediatrics, and pediatric hematology remained undecided.

**Fig. 2:**
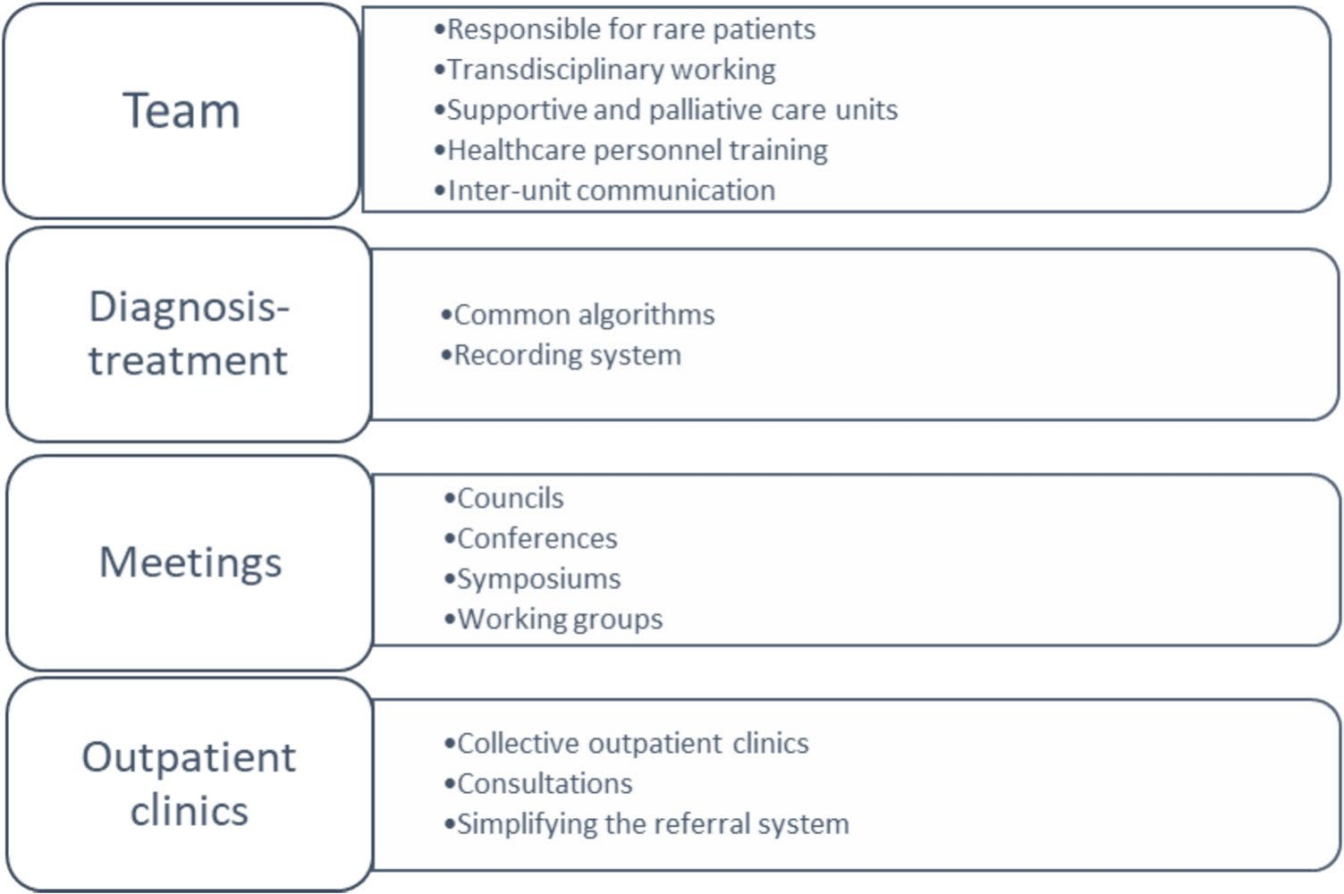
Recommendations for a multidisciplinary approach to rare diseases

Furthermore, all participants emphasized the necessity of a multidisciplinary approach in the management of rare diseases. However, 95% of the participants acknowledged encountering difficulties when implementing a multidisciplinary approach in their own clinical practice. The responses provided for suggestions on improving the multidisciplinary approach are detailed in Fig. 2.

**Fig. 3:**
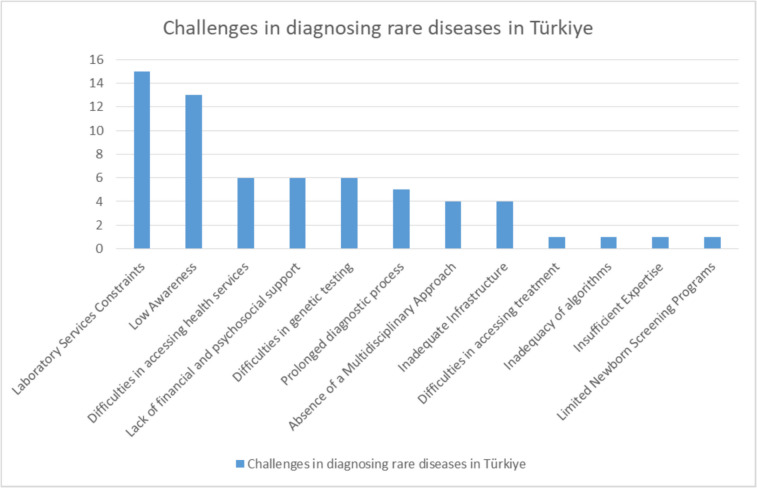
Challenges in diagnosing rare diseases in Türkiye

Critical factors among the most important challenges in diagnosing rare diseases in practice are shown in Fig. 3.

Table 3 delineates the advantages and disadvantages of clinics in the diagnosis and treatment of rare diseases.

**Table 3:** Advantages and disadvantages of the institution

Advantages	Disadvantages
Serving as a reference center with heightened awareness for rare diseases The presence of a multidisciplinary team and the facilitation of rapid communication between team members Availability of comprehensive laboratory facilities Easy access to an experienced team, even during on-call conditions Presence of research laboratories and support for scientific studies	Insufficiency of outpatient clinics and service areas Absence of integrated multidisciplinary outpatient clinics Deficiency in technological and logistical infrastructure Diagnostic tests are restricted in availability financially burdensome and require prolonged processing times Insufficient number of healthcare practitioners and auxiliary healthcare staff Overwhelming outpatient clinic workload resulting in fatigued physicians Restricted time allocated to patients with rare diseases Inadequate provision of psychosocial support teams for both patients and their families

To enhance the diagnosis and treatment of rare diseases, it has been deemed essential to improve patient services, develop research and development (R&D) projects, and strengthen production infrastructures. It was also stated that the support of state insurance was important. Moreover, there is a need to establish multidisciplinary approaches, implement preventive medicine strategies, and formulate comprehensive social policies.

Participants outlined several expectations from other departments within the clinic during the diagnostic and treatment processes. These include the establishment of collective outpatient clinics, the development of a common registration system, and the regular organization of multidisciplinary councils. Furthermore, there is a demand for advanced and rapid biochemical, genetic, and microbiological diagnostic services. Additionally, the development of strong clinical-laboratory partnerships, promoting a harmonious relationship based on mutual respect, understanding, and ensuring prompt communication between clinics were underscored as essential. The initiation of joint research endeavors was also highlighted as a significant expectation.

The clinic’s capability in delivering accurate diagnoses for rare diseases and providing appropriate treatments received a highly favorable assessment, with 48% of participants rating it as “good” and the remaining 52% as “very good.”

Recommendations for further enhancing the clinic’s diagnosis and therapeutic approaches to rare diseases included the following:

i. Establishment of rare disease centers or collaborative outpatient clinics: creating specialized centers or joint outpatient clinics dedicated to the comprehensive management of rare diseases
ii. Awareness training: implementing extensive training programs to enhance awareness among healthcare professionals and the general public regarding rare diseases
iii. Multi-center communication network: developing a robust communication network to facilitate seamless collaboration between multiple healthcare centers specializing in rare diseases
iv. National Registration System: establishing a centralized national registry to systematically record and track rare diseases cases, enhancing data collection and patient follow-up
v. Diagnosis-treatment-monitoring algorithms: designing standardized algorithms to streamline the diagnosis, treatment, and monitoring processes for rare diseases
vi. Access to advanced laboratory tests: expanding accessibility to advanced laboratory diagnostics essential for the accurate identification of rare diseases.
vii. Facilitation of treatment access: strengthening systems to ensure timely and equitable access to necessary treatments for rare disease patients
viii. Multidisciplinary research projects: promoting and conducting interdisciplinary research projects that address the multifaceted challenges posed by rare diseases
ix. Enhancement of physical conditions: upgrading the physical conditions of healthcare facilities to better meet the unique needs of rare disease patients
x. Support for healthcare personnel and equipment: providing sufficient support in terms of specialized healthcare personnel and cutting-edge equipment to improve service the quality of care
xi. Psychosocial and rehabilitation support: offering comprehensive psychosocial and rehabilitation support services to patients and their families, addressing the broader emotional, psychological, and social impact of living with rare diseases

## Discussion

Patients with rare diseases and their families encounter a multitude of challenges in diagnosis, treatment, and care, across all countries. Consequently, various nations have developed national strategic action plans for rare diseases. The Australian Government launched the country’s first National Strategic Action Plan for Rare Diseases in February 2020 [8]. Similarly, Japan, despite its rare disease policies dating back to the 1970s, only enacted new laws to aid these patients in 2014 [9]. In our country, the “2023–2027 Rare Diseases Health Strategy Document and Action Plan” was published in 2022 to address this pressing issue [6].

Approximately 72% of all rare diseases are attributable to genetic causes [5]. Our study, when querying departments about the rare diseases they most frequently encounter, revealed that the majority were genetic diseases (Table 2). Departments frequently encountering rare diseases in daily practice (81–100%) include medical genetics, inborn metabolic diseases, and pediatric hematology, primarily dealing with genetically induced diseases and cancers. In November–December 2019, the Economist Intelligence Unit conducted a survey among 503 healthcare workers across five Asia–Pacific countries to assess their understanding of rare diseases and identify challenges within national healthcare systems [10]. Remarkably, 14% of healthcare professionals surveyed (including 10% of specialists) reported never having seen a patient with a rare disease in their careers. Contrastingly, all participants in our survey had encountered a patient with a rare disease, though it should be noted that our sample size was smaller and all participants were affiliated with a university hospital.

In our study, all participants acknowledged the difficulty of diagnosing rare diseases, with 90% indicating that this challenge significantly impacted their daily practice. This finding aligns with the Economist Intelligence Unit survey, where participants also identified diagnostic difficulty as the primary issue in rare diseases [10].

In Europe, registration systems for rare diseases have been established for various purposes, including defining the natural history and phenotypic diversity of rare diseases, improving treatment indications, devising risk stratification strategies, and developing disease-specific guidelines [1]. Consistently, 85% of our participants advocated for a distinct registration system for rare diseases.

The importance of a multidisciplinary approach in the diagnosis and management of rare diseases is well-recognized. In 2015, UD-PrOZA (Program for Undiagnosed Rare Diseases) was founded at the Ghent University Hospital in Belgium to facilitate the diagnostic process for adult patients with undiagnosed rare diseases [11]. This multidisciplinary initiative resulted in definitive diagnoses for 18% of patients. In various countries, similar single-center or multi-center multidisciplinary teams have been established, achieving a definitive diagnosis rate of up to 67% [12]. Our survey results align with these findings, with all participants advocating for a multidisciplinary approach in the management of rare diseases. However, the majority acknowledged significant challenges in translating this approach into routine clinical practice. Key criteria for the successful implementation of multidisciplinary teams include the formal establishment of the team, regular joint meetings, and the integration of joint outpatient clinic services.

Diagnosing rare diseases presents major challenges due to their low prevalence, heterogeneous clinical manifestations, and often limited awareness within the medical community. A multidisciplinary approach significantly improves diagnostic accuracy by harnessing a wide array of specialized expertise and advanced diagnostic methodologies. This model facilitates comprehensive patient evaluation through holistic assessments and regular interdisciplinary case discussions, enabling the development of personalized treatment plans tailored to the specific needs of each patient. Moreover, early and precise diagnoses, supported by continuous monitoring, contribute to better patient outcomes. In addition to improving clinical care, a multidisciplinary approach fosters advancements in medical knowledge through collaborations with research institutions and enhances education and awareness, within the medical community. It also provides critical support to patients and their families by offering genetic counseling and comprehensive psychosocial services. This collaborative framework ensures that patients with rare diseases receive optimal care while deepening the overall understanding and management of these complex conditions.

Beyond the absence of a multidisciplinary approach, other substantial challenges in diagnosing rare diseases include limited awareness and a shortage of experienced, specialized healthcare professionals. These interconnected issues highlight the critical need for a comprehensive strategy aimed at improving the diagnosis and management of rare diseases. Such a strategy should encompass raising awareness among both healthcare providers and the general public, strengthening infrastructure, to support rare disease diagnosis and care, enhancing the training and expertise of medical professionals, and establishing robust support systems for patients and families. Awareness training was among the solutions proposed by participants to improve rare disease diagnosis and treatment. For example, at the Necker-Cochin Faculty of Medicine in Paris, all third-year medical students are offered an optional 30-h training course in rare diseases, supplementing routine genetics training [13]. Elliot et al. suggested that similar courses could be implemented in Australia to aid general practitioners and other clinicians in the early diagnosis and management of rare diseases [14].

The capability of our clinic to accurately diagnose rare diseases and administer appropriate treatment was evaluated positively, with 50% rating it as good and 50% as very good, attributable to the clinic’s inherent advantages (Table 3). However, it is acknowledged that patients with rare diseases in rural areas of our country have a much lower chance of receiving timely diagnosis and treatment.

This study aimed to highlight the challenges and solutions experienced by experts dealing with rare diseases at a university hospital in Türkiye. Our study is the first of its kind conducted in Türkiye, and the expert opinions on the problems and potential solutions for rare disease patients in our country will contribute to the literature and inspire further studies with larger participant groups.

In conclusion, this study highlights significant challenges and potential solutions for the diagnosis and treatment of rare diseases in the clinical practice. It is the first study of its kind in our country, where a wide range of rare diseases are observed. Despite advances in medical technology, the most critical barriers to diagnosing, treating, and monitoring rare diseases remain inadequate laboratory infrastructure and low awareness among healthcare professionals. Addressing these challenges requires a multifaceted approach, including increasing awareness of rare diseases, enhancing medical training, strengthening R&D and production capacities, fostering multidisciplinary collaboration, and implementing preventive healthcare and supportive social policies.

## Data Availability

Data sets generated during the current study are available from the corresponding author on reasonable request.

## Abbreviations

WHO: World Health Organization
IEM: Inborn error of metabolism
R&D: Research and development
UD-PrOZA: Program for Undiagnosed Rare Diseases
